# Foliar application of salicylic acid inhibits the cadmium uptake and accumulation in lettuce (*Lactuca sativa* L.)

**DOI:** 10.3389/fpls.2023.1200106

**Published:** 2023-08-11

**Authors:** Wen Tang, Le Liang, Yongdong Xie, Xiaomei Li, Lijin Lin, Zhi Huang, Bo Sun, Guochao Sun, Lihua Tu, Huanxiu Li, Yi Tang

**Affiliations:** ^1^ College of Horticulture, Sichuan Agricultural University, Chengdu, Sichuan, China; ^2^ Institute for Processing and Storage of Agricultural Products, Chengdu Academy of Agriculture and Forestry Sciences, Chengdu, Sichuan, China; ^3^ Vegetable Germplasm Innovation and Variety Improvement Key Laboratory of Sichuan, Sichuan Academy of Agricultural Sciences, Chengdu, Sichuan, China; ^4^ Rice and Sorghum Research Institute, Sichuan Academy of Agricultural Sciences, Deyang, Sichuan, China; ^5^ College of Forestry, Sichuan Agricultural University, Chengdu, Sichuan, China

**Keywords:** salicylic acid, lettuce, cadmium, ion transporter gene, antioxidant enzyme

## Abstract

**Introduction:**

Salicylic acid (SA) is a multi-functional endogenous phytohormone implicated in the growth, development, and metabolism of many plant species.

**Methods:**

This study evaluated the effects of different concentrations of SA (0, 25, 100, 200, and 500 mg/L) on the growth and cadmium (Cd) content of lettuce (*Lactuca sativa* L.) under Cd stress. The different concentrations of SA treatments were administered through foliar application.

**Results:**

Our results showed that 100-200 mg/L SA significantly increased the plant height and biomass of lettuce under Cd stress. When SA concentration was 200 mg/L, the plant height and root length of lettuce increased by 19.42% and 22.77%, respectively, compared with Cd treatment alone. Moreover, 200 mg/L and 500mg/L SA concentrations could reduce peroxidase (POD) and superoxide dismutase (SOD) activities caused by Cd stress. When the concentration of exogenous SA was 500 mg/L, the POD and SOD activities of lettuce leaves decreased by 15.51% and 19.91%, respectively, compared with Cd treatment. A certain concentration of SA reduced the uptake of Cd by the lettuce root system and the transport of Cd from the lettuce root system to shoots by down-regulating the expression of *Nramp5*, *HMA4*, and *SAMT*, thus reducing the Cd content of lettuce shoots. When the concentration of SA was 100 mg/L, 200 mg/L, and 500 mg/L, the Cd contents of lettuce shoots were 11.28%, 22.70%, and 18.16%, respectively, lower than that of Cd treatment alone. Furthermore, principal component and correlation analyses showed that the Cd content of lettuce shoots was correlated with plant height, root length, biomass, antioxidant enzymes, and the expression level of genes related to Cd uptake.

**Discussion:**

In general, these results provide a reference for the mechanism by which SA reduces the Cd accumulation in vegetables and a theoretical basis for developing heavy metal blockers with SA components.

## 1 Introduction

Cadmium (Cd) is a nonessential element for plants, and it causes plant poisoning when its toxicity threshold is exceeded (Saidi et al., 2014). Cd plant poisoning symptoms include yellowing and chlorosis of leaves, poor plant development, restricted root growth, photosynthesis inhibition, changes in chloroplast ultrastructure, lipid peroxidation, and nitrogen metabolism disorders (Ran et al., 2015; Haider et al., 2021). These symptoms often reduce crop yield (Aziz et al., 2015). Khan et al. showed that 50 μmol/L of Cd significantly reduced the biomass of *Brassica rapa ssp. chinensis* L. and reduced leaf photosynthetic parameters (photosynthetic rate, stomatal conductance, transpiration rate, and intercellular CO_2_ concentration) (Khan et al., 2020). In addition, Cd can be transferred and accumulated in the edible part of crops, directly endangering human health through the food chain (Wang et al., 2019). Several studies showed that exogenous plant hormones and other antioxidant substances could activate the defense mechanism of plants to reduce Cd toxicity on plants (Chen et al., 2020; Waheed et al., 2021; Wang et al., 2021a). Moreover, spraying exogenous substances is an economical, effective, and easy measure of alleviating the Cd uptake and stress on crops. Salicylic acid (SA), a small molecular phenolic substance widely existing in plants, and participates in the growth and development and various physiological and biochemical activities in plants and can activate stress-related resistance metabolism in plants (Metwally et al., 2003; Sharma et al., 2020). Previous studies showed that SA could be a key signal molecule to mediate plant responses to biotic and abiotic stresses, including drought, salinity, cold, osmotic, and heavy metal stress (Szepesi et al., 2009; Khan et al., 2015). Several studies also showed that the exogenous application of SA can effectively alleviate the toxic effects and reduce the accumulation of Cd in plants under Cd stress (Metwally et al., 2003; Singh et al., 2015; Wang et al., 2021d). For example, Guo et al. (2007)showed that SA pretreatment could alleviate Cd-mediated inhibition on the growth of rice roots and enhance the antioxidant activity of rice under Cd stress. This reduced the toxicity induced by Cd and enhanced the rice tolerance to Cd. Similarly, Li et al. (2019) reported that spraying potato leaves with 600 μmol/L SA could alleviate the toxic effect of Cd (200 μmol/L Cd) by increasing the relative water content, chlorophyll, proline, and endogenous SA content of leaves and stimulating the antioxidant enzyme activity. Wang et al. also showed that spraying SA could reduce Cd accumulation in rice by regulating the expression level of genes related to Cd transport and absorption (*OsNramp1*, *OsNramp5*, *OsHMA2*, *OsHMA3*, and *OsHMA9*) (Wang et al., 2021c). However, there are relatively few reports on how SA changes the physiological mechanisms of Cd transport and accumulation in leafy vegetables.

Leafy vegetables are an indispensable part of a healthy diet. Leafy vegetables are more sensitive to Cd pollution than other crops and vegetables (such as Solanaceae, cabbage, root vegetables, onions, legumes, etc.) (Xiao et al., 2018; Wang et al., 2021e). Among them, lettuce (*Lactuca sativa* L.) is rich in various nutrients and highly edible. Though very sensitive to soil Cd pollution, lettuce is one of the leafy vegetables with the highest Cd accumulation ability, which greatly increases the risk of Cd entering the human body (Mehmood et al., 2013). Tang et al. found that 50 μmol//L cadmium significantly reduced the growth indicators of lettuce and significantly accumulated Cd content in the shoot, and caused stress but not death (Tang et al., 2022). However, there is insufficient information on the effects of different concentrations of SA on leafy vegetables under Cd stress conditions. Therefore, this study investigated the effects of exogenous spraying of the same concentration of SA on lettuce physiology, biochemistry, cadmium content, and gene expression related to SA synthesis under 50μmol/L Cd stress. To provide a good reference basis for improving the cadmium resistance of lettuce and ensuring the safe production of vegetables.

## 2 Materials and methods

### 2.1 Materials

The test species ‘Glass lettuce’ seeds were purchased from the Chengdu seed station (Chengdu, China). The Hoagland nutrient solution was used for cultivation, and the Cd compound was CdCl_2_·2.5H_2_O (analytical grade). SA was obtained from Sigma-Aldrich (St. Louis, MO, USA).

### 2.2 Experimental design

The study was conducted at the Chengdu Campus of the Sichuan Agricultural University from February to June 2022. Full and uniform-sized lettuce seeds were sterilized in 10% (v/v) hydrogen peroxide (H_2_O_2_) for 10 min, rinsed with ultrapure water, and evenly distributed in petri dishes containing moist filter papers. The plates were then placed in a 20 °C artificial incubator for germination. Thereafter, sprouted seeds were seeded in a seedling tray containing perlite and vermiculite in a 1:1 ratio and transferred to an artificial incubator. Half-strength Hoagland nutrient solution was added to the tray at an appropriate time daily, and the incubator was set to 23/18 °C (day/night) and a 14/10 h (day/night) photoperiod under a 200 μmol/m^2^/s light intensity. When four true leaves of the seedlings had fully unfolded, seedlings with vigorous and consistent growth were selected and transplanted into a 10×10 cm (depth × height) nutrient container with equal volumes of perlite and vermiculite. Each container contained one plant, and the containers were placed in a plastic dish (with a height of 8 cm) filled with full-strength Hoagland’s nutrient solution, which was replaced every 3 days.

Three days after seedling transplantation, the lettuce leaves treated with CK (the control without Cd) and Cd treatments were sprayed with distilled water and different concentrations of SA solution. After 3 days of pretreatment, all treatment groups other than CK were treated with Hoagland nutrient solution containing 50 μmol/L Cd and different concentrations of SA solution. Similar to the pretreatment, the treatments were sprayed once every three days, for a total of three times. Throughout the experiment, different concentrations of SA solution were sprayed four times (pretreatment once and treatment thrice after adding Cd). When replacing the nutrient solution, we ensured that at least 30% of the nutrient solution flowed out to prevent Cd accumulation in the nutrient bowl. The six experimental treatments applied in the experiment included: CK (the control without Cd), Cd (50 μmol/L Cd), Cd + SA 25 (50 μmol/L Cd + 25 mg/L SA), Cd + SA 100 (50 μmol/L Cd +100 mg/L SA), Cd + SA 200 (50 μmol/L Cd + 200 mg/L SA), and Cd + SA 500 (50 μmol/L Cd + 500 mg/L SA). Throughout the experiment, the temperature was 23/18 °C (day/night), with a relative humidity of 75% – 80%, a light cycle of 14/10 h (day/night), and a light intensity of 300 μ mol/m^2^/s in the artificial culture room. The pots were haphazardly rearranged regularly to weaken the impact of edge effects and promote the timely prevention and control of pests and diseases.

### 2.3 Sample analysis

Lettuce samples were collected 10 days after the last spraying of exogenous SA treatment, and the growth and morphological indicators of the whole plants were measured. Thereafter, fresh root and shoot samples were freeze-dried in liquid nitrogen and stored at -80 °C in an ultra-low temperature refrigerator for the subsequent determination of physiological and quality indicators. For the Cd content determination, shoot and root samples were fixed in an oven at 105 °C for 15 min and then dried at 75 °C to a constant weight.

#### 2.3.1 Determination of plant growth and biomass

The plant height and root length of lettuce plants were measured to the nearest millimeter using a ruler. Thereafter, the lettuce shoots and roots were washed with tap water and rinsed with deionized water thrice, followed by oven-drying at 105 °C for 15 min and at 75 °C to constant weight for dry biomass determination.

#### 2.3.2 Determination of photosynthetic pigment content

The second and third functional leaves from the shoot tip (n = 3) were used to determine the contents of photosynthetic pigments (chlorophyll a, chlorophyll b, and carotenoid) using the ethanol and acetone extraction methods described previously (Tang et al., 2020).

#### 2.3.3 Determination of photosynthetic parameters

The same leaves were used to determine the net photosynthetic rate (Pn), stomatal conductance (Gs), transpiration rate (Tr), and intercellular CO_2_ concentration (Ci) with a LI-6400XT portable photosynthetic system (LI-COR Inc., Lincoln, NE). The photosynthetic parameters were manually set at 25 °C, 1000 μmol/m^2^/s light intensity, and a CO_2_ concentration of 400 μmol/mol (Tang et al., 2022). Stomatal limitation (Ls) was 1-Ci/Ca, where Ca is the atmospheric CO_2_ concentration.

#### 2.3.4 Determination of membrane peroxidation

The proline content was assayed using the sulfosalicylic acid method, while the soluble protein and sugar contents were assayed using the Coomassie brilliant blue G-250 anthrone–ethyl acetate methods, respectively. The relative conductivity was assayed using a conductivity meter (Hi-Fidelity Technology Co. Ltd.; Beijing, China), and all the assays were conducted according to the methods described by Wang et al. (2021b).

#### 2.3.5 Determination of antioxidant enzyme indexes

The antioxidant enzyme activity assays were conducted as previously described (Liang et al., 2022). Superoxide dismutase (SOD) activity was measured using the nitroblue tetrazolium method, peroxidase (POD) activity using the guaiacol method, and catalase (CAT) activity using the ultraviolet (UV) absorption method.

#### 2.3.6 Determination of cadmium content

Plant samples (0.5 g) were treated with a 4:1 nitric acid: perchloric acid solution (v:v) for 12 h, digested to a clear solution, filtered, and diluted to a volume of 50 mL. The Cd content was then determined using an iCAP 6300 ICP spectrometer (Thermo Scientific, Waltham, MA, USA) (Tang et al., 2020). The translocation factor (TF) was calculated as the Cd content of shoots divided by the Cd content of roots (Rastmanesh et al., 2010).

#### 2.3.7 Determination of the relative expression of genes related to cadmium uptake and SA synthesis in lettuce

The total RNA was extracted from all samples using a TIANGEN Biotech Co., Ltd. RNA prep pure plant kit (TIANGEN Biotech Co., Ltd., Beijing, China), according to the manufacturer’s instructions. Real-time fluorescence quantitative PCR was conducted on a real-time quantitative PCR instrument (CFX Connect; Bio-Rad, Hercules, CA, USA) using 2X M5 HiPer SYBR Premix EsTaq (with Tli RNaseH) (Mei5 Biotechnology Co., Ltd.). All primers were synthesized by Beijing Tsingke Biotechnology Co., Ltd. (Beijing, China) and are listed in **Table 1**. The relative expression level of the genes was calculated by the 2^−ΔΔCT^ method. Three independent biological replicates were set per sample (Pan et al., 2021).

**Table 1:** Primer information of quantitative real-time PCR.

Gene name	Gene symbol in NCBI	Forward primer(5’-3’)	Reverse Primer(5’-3’)
*Nramp2*	LOC111889112	CTCCGGTCGTCAACTCTTCC	ATCTTCGTCATCCGAGGTGC
*Nramp5*	LOC111901958	AGCTATGTGAAGCCACCAGC	GCATGCATCGTTGACACCAT
*HMA3*	LOC111887653	GGGGTGTTCACAAGTTCCCA	TGTGCGAAATTGGCTGCTTC
*HMA4*	LOC111881074	GCTTTGGAGTAGGAATGGAAGT	GTGGTGACACAATGGCACTTT
*WRKY6*	LOC111907870	TCGAGCAAGCTAATGACCCC	TGTGGGTGCCTTCATAGGTG
*SAMT*	LOC111890312	TTGTTCGCCGGAGAAACGTA	CGGTATGCCCTTGTGTCCAT
*Actin*		GTGAGTGAAGAAGGGCAATG	CACTTTCAACCCGATTCACC

### 2.4 Statistical analyses

Data were compiled and organized using Excel 2016 software (Microsoft Corp., Redmond, WA, USA), and statistical analyses were conducted using SPSS 25.0 statistical software (IBM, Armonk, NY, USA). The results were analyzed by one-way ANOVA with Duncan’s multiple range test at the *P* < 0.05 significance level and presented as the means of three biological replicates ± standard error (SE). We conducted principal component and correlation analyses to study the relationship between various indicators. The figures were constructed using Origin Pro 2021 software (Electronic Arts Inc, USA).

## 3 Results

### 3.1 Plant growth and biomass

**Figure 1:**
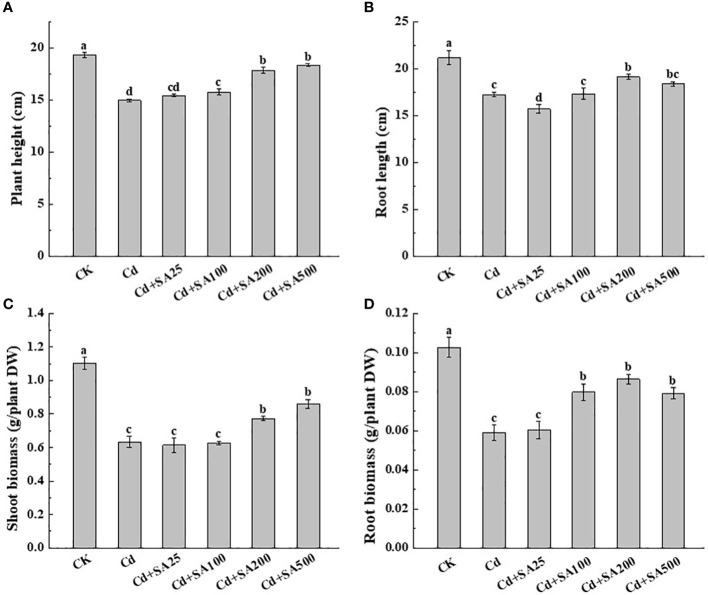
Effects of different concentrations of exogenous SA on lettuce growth and biomass under Cd stress. **(A)** The plant height. **(B)** The root length. **(C)** The shoot biomass of dry weight. **(D)** The root biomass of dry weight. CK: control; Cd: 50μmol/L Cd; Cd+SA25: 50μmol/L Cd+25mg/L SA; Cd+SA100: 50μmol/L Cd+100mg/L SA; Cd+SA200: 50μmol/L Cd+200mg/L SA; Cd+SA500: 50μmol/L Cd+500mg/L SA. Data are means ± SE of 3 replicate samples. Values with the different letters are significantly different (*P* < 0.05).

Cd stress significantly reduced the plant height, root length, shoot biomass, and root biomass of lettuce by 22.63%, 18.58%, 42.60%, and 42.53%, respectively, compared with CK (**Figure 1**). The plant height, root length, shoot biomass, and root biomass of lettuce treated with 200 and 500 mg/L SA were significantly higher and increased by 19.42%, 22.77%, 10.83%, and 6.58%, respectively compared to those treated with Cd treatment alone (**Figures 1A, B**). In addition, the shoot and shoot biomass of lettuce increased with the increase of SA concentration under Cd stress (**Figures 1C, D**). When SA concentration was 500 mg/L, the shoot and shoot biomass increased by 35.79% and 33.90%, respectively, compared with Cd treatment alone (**Figures 1C, D**).

### 3.2 Photosynthetic pigment content

**Figure 2:**
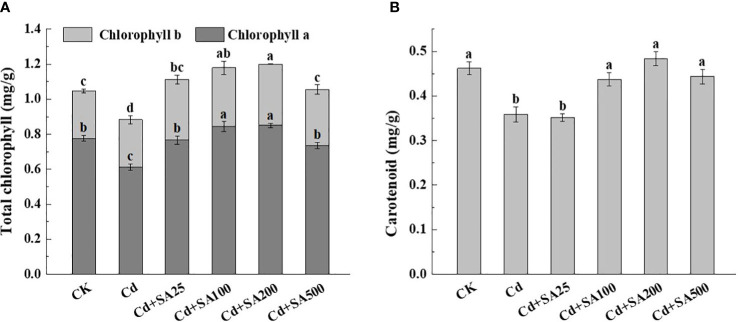
Effects of different concentrations of exogenous SA on the photosynthetic pigment content of lettuce under Cd stress. **(A)** The total chlorophyll content. **(B)** The carotenoid content. CK: control; Cd: 50μmol/L Cd; Cd+SA25: 50μmol/L Cd+25mg/L SA; Cd+SA100: 50μmol/L Cd+100mg/L SA; Cd+SA200: 50μmol/L Cd+200mg/L SA; Cd+SA500: 50μmol/L Cd+500mg/L SA. Data are means ± SE of 3 replicate samples. Values with the different letters are significantly different (*P* < 0.05).

Compared with CK, Cd stress significantly reduced the contents of chlorophyll and carotenoid in lettuce leaves (**Figure 2**). However, different concentrations of SA increased the content of chlorophyll a, chlorophyll b, and carotenoid in lettuce leaves compared with Cd stress (**Figure 2**). When SA concentration was 25, 100, 200, and 500 mg/L, the total chlorophyll content of leaves increased by 26.11%, 33.70%, 36.11%, and 19.54%, respectively compared with Cd treatment (**Figure 2A**). Similarly, when SA concentration was 100 mg/L and 200 mg/L, the chlorophyll a content of lettuce leaves increased by 37.49% and 38.55%, respectively, compared with Cd treatment alone (**Figure 2A**). The carotenoid content of lettuce increased with the increase of SA concentration, and when SA concentration was 200 mg/L, the carotenoid content of lettuce leaves increased by 34.80% compared with Cd treatment alone (**Figure 2B**).

### 3.3 Photosynthetic parameters

Cd stress significantly reduced the Pn and Ls of lettuce by 35.62% and 22.22% (**Table 2**) but significantly increased the Gs, Ci, and Tr of lettuce by 26.32%, 8.03%, and 60.49%, respectively, compared with CK. The different concentrations of exogenous SA could increase the Pn and Ls of lettuce; for example, when the concentration of SA was 200 mg/L, the Pn and Ls increased by 51.97% and 30.00%, respectively, compared with Cd treatment alone. However, exogenous SA reduced the Gs, Ci, and Tr of lettuce leaves to a certain extent, and when the concentration of SA was 100 mg/L, the Ci and Tr of lettuce leaves decreased by 9.70% and 29.23%, respectively, compared with Cd treatment alone (**Table 2**).

**Table 2:** The effect of SA treatment on the photosynthesis of lettuce under Cd stress.

Treatments	Pn (µmolm^-2^ s^-1^)	Gs (mol m^-2^ s^-1^)	Ci (µmol mol^-1^)	Tr (mmol m^-2^ s^-1^)	Ls
CK	13.39 ± 0.692a	0.19 ± 0.003b	293.12 ± 2.167b	1.62 ± 0.026d	0.27 ± 0.005b
Cd	8.62 ± 0.447c	0.24 ± 0.009a	316.65 ± 4.498a	2.60 ± 0.016a	0.21 ± 0.011c
Cd+SA25	9.89 ± 0.070c	0.22 ± 0.006a	299.39 ± 4.119b	2.50 ± 0.017b	0.25 ± 0.010b
Cd+SA100	11.31 ± 0.349b	0.20 ± 0.004b	285.95 ± 6.695bc	1.84 ± 0.014c	0.29 ± 0.017ab
Cd+SA200	13.10 ± 0.364a	0.23 ± 0.006a	278.64 ± 3.133bc	1.62 ± 0.024d	0.30 ± 0.008a
Cd+SA500	12.02 ± 0.499ab	0.19 ± 0.010b	288.44 ± 3.606c	1.87 ± 0.028c	0.28 ± 0.009ab

Ci, intercellular CO_2_ concentration; Gs, stomatal conductance; Pn, net photosynthetic rate; Tr, transpiration rate; Ls, stomatal limitation. CK: control; Cd: 50μmol/L Cd; Cd+SA25: 50μmol/L Cd+25mg/L SA; Cd+SA100: 50μmol/L Cd+100mg/L SA; Cd+SA200: 50μmol/L Cd+200mg/L SA; Cd+SA500: 50μmol/L Cd+500mg/L SA. Data are means ± SE of 3 replicate samples. Values with the different letters are significantly different (P < 0.05).

### 3.4 Membrane lipid peroxidation degree and osmotic regulating substances

Cd stress significantly increased the proline content, relative conductivity, and soluble protein content of lettuce leaves by 28.64%, 96.98%, and 32.96%, respectively, compared with CK (**Table 3**). With the increase of exogenous SA concentration, the soluble sugar content of lettuce leaves gradually increased; when SA concentration was 500 mg/L, the soluble sugar content increased by 27.97% compared with Cd treatment alone (**Table 3**). Conversely, exogenous SA significantly reduced the proline content of lettuce leaves under Cd stress; when the concentration of SA was 100 mg/L, the proline content decreased by 21.56% compared with Cd treatment alone. The exogenous SA concentration of 200 mg/L reduced the relative conductivity of lettuce leaves by 32.82% compared with the Cd treatment alone (**Table 3**).

**Table 3:** The effect of SA treatment on osmotic adjustment substances in lettuce under Cd stress.

Treatments	Proline (μg/g)	Relative Conductivity(%)	Soluble sugar Content (% FW)	Soluble protein Content (mg/g FW)
CK	33.03 ± 0.084d	6.96 ± 0.285c	1.35 ± 0.011a	13.50 ± 0.162e
Cd	42.49 ± 0.438a	13.71 ± 1.009a	0.85 ± 0.023e	17.95 ± 0.022b
Cd+SA25	33.44 ± 0.592d	10.22 ± 0.434b	0.96 ± 0.008d	18.93 ± 0.071a
Cd+SA100	33.33 ± 0.607d	8.69 ± 0.325bc	0.95 ± 0.016d	17.80 ± 0.086b
Cd+SA200	39.61 ± 0.356b	7.27 ± 0.435c	1.05 ± 0.024c	16.02 ± 0.121c
Cd+SA500	36.58 ± 0.172c	9.21 ± 0.492b	1.18 ± 0.009b	14.80 ± 0.078d

CK: control; Cd: 50μmol/L Cd; Cd+SA25: 50μmol/L Cd+25mg/L SA; Cd+SA100: 50μmol/L Cd+100mg/L SA; Cd+SA200: 50μmol/L Cd+200mg/L SA; Cd+SA500: 50μmol/L Cd+500mg/L SA. Data are means ± SE of 3 replicate samples. Values with the different letters are significantly different (P < 0.05).

### 3.5 Antioxidant enzyme activities

**Figure 3:**
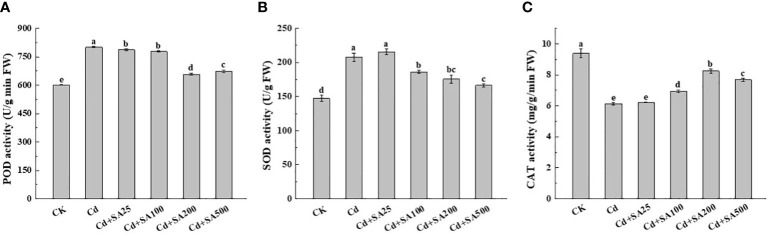
Effects of different exogenous SA on the activity of antioxidant enzymes in lettuce under Cd stress. **(A)** POD activity, **(B)** SOD activity, **(C)** CAT activity. CK: control; Cd: 50μmol/L Cd; Cd+SA25: 50μmol/L Cd+25mg/L SA; Cd+SA100: 50μmol/L Cd+100mg/L SA; Cd+SA200: 50μmol/L Cd+200mg/L SA; Cd+SA500: 50μmol/L Cd+500mg/L SA. Data are means ± SE of 3 replicate samples. Values with the different letters are significantly different (*P* < 0.05).

Cd stress significantly increased the POD and SOD activities of lettuce leaves by 33.23% and 41.19%, respectively, compared with CK (**Figures 3A, B**). However, Cd stress significantly reduced the CAT activity of lettuce leaves by 34.64% lower than that of CK (**Figure 3C**). Different concentrations of exogenous SA significantly reduced the POD and SOD activities of lettuce leaves compared with Cd treatment alone. The POD and SOD activities of lettuce leaves decreased by 17.85% and 15.87%, respectively, when the concentration of exogenous SA was 200 mg/L and by 15.51% and 19.91%, respectively, when the concentration of exogenous SA was 500 mg/L, compared with Cd treatment alone (**Figures 3A, B**). In addition, exogenous SA could significantly increase the CAT activity of lettuce leaves under Cd stress; when SA concentration was 200 mg/L, the CAT activity of lettuce leaves increased by 34.46% compared with Cd treatment alone (**Figure 3C**).

### 3.6 Cadmium content

**Figure 4:**
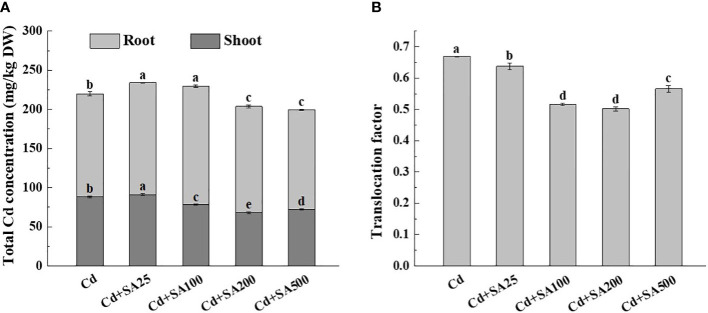
Effects of different exogenous SA on Cd content and Cd translocation factor of lettuce under stress. **(A)** Cd concentration, **(B)** Translocation factor. Cd: 50μmol/L Cd; Cd+SA25: 50μmol/L Cd+25mg/L SA; Cd+SA100: 50μmol/L Cd+100mg/L SA; Cd+SA200: 50μmol/L Cd+200mg/L SA; Cd+SA500: 50μmol/L Cd+500mg/L SA. Data are means ± SE of 3 replicate samples. Values with the different letters are significantly different (*P* < 0.05).

Under Cd stress, the total Cd content of lettuce increased initially and then decreased with the increase of exogenous SA concentration. When SA concentration was 200 mg/L and 500 mg/L, the total Cd content of lettuce decreased by 7.29% and 9.22%, respectively, compared with Cd treatment alone (**Figure 4A**). Similarly, when the concentration of SA was 100 mg/L, 200 mg/L, and 500 mg/L, the Cd content in the lettuce shoots was 11.28%, 22.70%, and 18.16%, respectively, lower than that under Cd treatment alone (**Figure 4A**). The SA concentrations of 100 mg/L and 200 mg/L also reduced the translocation factor of Cd from lettuce roots to shoots by 22.80% and 24.96%, respectively, compared with Cd treatment alone (**Figure 4B**).

### 3.7 Expression of genes responsible for Cd uptake and transport and SA synthesis

**Figure 5:**
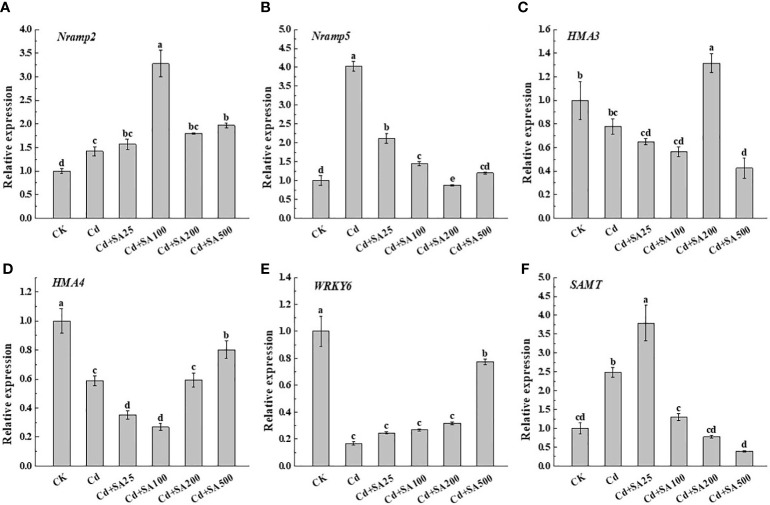
Effects of different exogenous SA on gene expression of *Nramp2* **(A)**, *Nramp5* **(B)**, *HMA3* **(C)**, *HMA4* **(D)**, *WRKY6* **(E)**, *SAMT* **(F)** in lettuce under Cd stress. Values are means ± SE from three independent experiments. Values with different letters above the bars are significantly different at *P*<0.05 according to LSD.

Compared with CK, Cd stress significantly increased the relative expression levels of *Nramp2*, *Nramp5*, and *SAMT* by 42.00%, 302.84%, and 148.17%, respectively (**Figures 5A, B, F**). However, Cd stress significantly down-regulated the relative expression levels of *HMA3*, *HMA4*, and *WRKY6* compared to CK (**Figures 5C, D, E**). Under Cd stress, exogenous SA could up-regulate the relative expression of *HMA3* and *HMA4*, and when SA concentration was 200 mg/L, the relative expression of *HMA3* increased by 68.76% compared with Cd treatment alone (**Figures 5F, E**). When the concentration of SA was 25 mg/L, the relative expression of *SAMT* increased by 279.10% compared with CK and by 52.76% compared with Cd treatment alone (**Figure 5F**). These results showed that SA could alleviate Cd stress on lettuce by regulating the Cd absorption and transportation and the expression of genes related to SA synthesis in lettuce.

### 3.8 Principal component analysis

**Figure 6:**
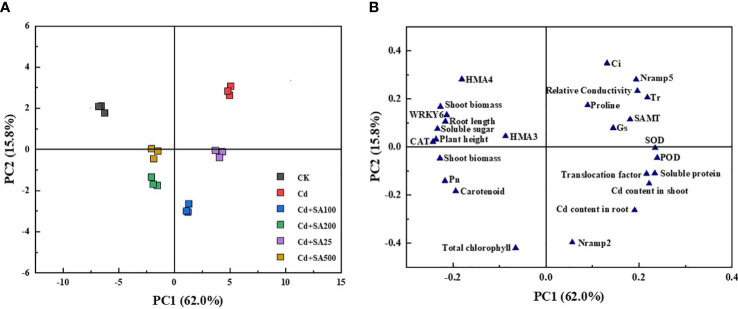
Principal component analysis of lettuce growth, physiological indexes and Cd content under Cd stress by different exogenous SA treatment. **(A)** PCA score plot; **(B)** PCA loading plot.

Principal component analysis (PCA) was performed to characterize the changes in various lettuce indicators under Cd stress conditions and different concentrations of SA treatment. The results showed that PCA could clearly separate lettuce samples from different treatments, and there were significant differences between the treatments (**Figure 6A**). The first component (PC1) and the second component (PC2) explained 62.0% and 15.8% of the variance, respectively, and the cumulative variance interpretation rate was 77.9%. PC1 was positively correlated with the shoot and root Cd contents, the translocation factor, and the relative expression of *Nramp2* in lettuce but negatively correlated with the shoot and root biomass and the relative expression of *HMA3* and *HMA4* in lettuce (**Figure 6B**). Moreover, total chlorophyll content, carotenoid content, and Pn were negatively correlated with PC2, indicating that exogenous Cd and SA significantly impacted the photosynthesis of lettuce.

### 3.9 Correlation analysis

**Figure 7:**
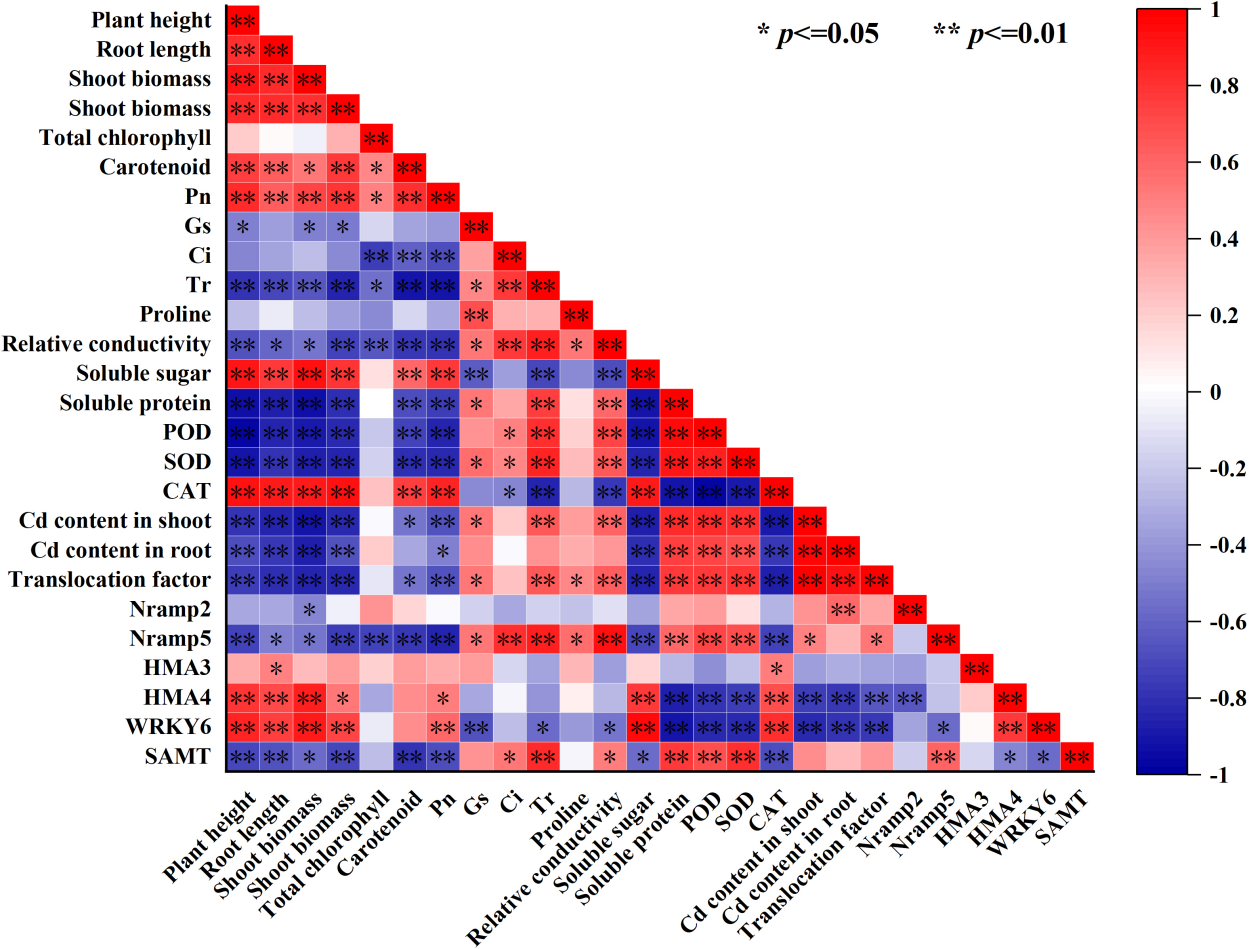
Correlation analysis of lettuce growth, physiological indexes and Cd content under Cd stress by different exogenous SA treatment.

To further intuitively display the relationship between the various lettuce indicators, we conducted a correlation analysis based on the heat map, as shown in **Figure 7**, the shoot and root Cd content and the Cd translocation factor in the lettuce had a significant positive correlation with the soluble protein, POD activity, and SOD activity but a negative correlation with the plant height (A), root length, shoot biomass, root biomass, soluble sugar, CAT activity and relative expression of *WRKY6*, and *HMA4* (*p* ≤ 0.01) (**Figure 6**). At the same time, there was no significant correlation between Cd content in the shoot and root of lettuce, as well as Cd transport factors, and total chlorophyll content, Ci, and proline content in leaves (**Figure 7**). Moreover, carotenoid content had a significant positive correlation with plant height, root length, root biomass, soluble sugar, and CAT activity (*P* ≤ 0.01), but not significantly correlated with the relative expression of *WRKY6*, *HMA3*, *HMA4* and *Nramp2* (**Figure 5**). The relative expression of *Nramp2* and *HMA3* were not significantly correlated with other indicators, while the relative expression of *HMA*4 and *WRKY6* positively correlated with plant height, root length, shoot biomass, soluble sugar, and CAT activity. Moreover, the relative expression of *HMA4* and *WRKY6* was positively correlated with plant height, root length, shoot biomass, solve sugar, and CAT activity but negatively correlated with SOD activity, shoot and root Cd content, and Cd translocation factor of lettuce (*p* ≤ 0.01) (**Figure 6**). Contrary to *HMA4* and *WRKY6*, the relative expression of *SAMT* had a significant negative correlation with plant height, root length, root biomass, carotenoid, and Pn, and a significant positive correlation with soluble protein, POD activity, SOD activity, and the relative expression of *Nramp5* (*p* ≤ 0.01).

## 4 Discussion

Cd stress seriously affects the normal growth and development of most crops, resulting in symptoms such as chlorosis and yellowing of leaves, curling of leaves, delayed growth of stems, reduction of lateral roots, root tip necrosis, and plant dwarfing (Haider et al., 2021; Shaari et al., 2022). In this study, our results show that 50 μmol/L Cd significantly inhibited the growth of lettuce and reduced its biomass, consistent with the results of previous studies (Lavres et al., 2019; Huang et al., 2022b). Excessive concentration of salicylic acid can seriously damage the growth and development of plants. Research has shown that low concentration of salicylic acid can maintain a high electron transfer rate in the chloroplasts of tomato Guard cell, while high concentration of salicylic acid can destroy the photosynthetic electron transfer (Lawson, 2009). In our preliminary experiments, we found that high concentrations of SA would cause osmotic stress in leaves, leading to wilting. Therefore, we chose 25-500 mg/L concentrations of exogenous SA for lettuce treatment under Cd stress. Previous studies showed that 25 μmol/L of SA could alleviate Cd-mediated inhibition on rice growth and increase rice biomass (Majumdar et al., 2020). Moreover, Krantev et al. found that SA pretreatment of maize (*Zea mays* L.) seeds could reduce the negative impact of Cd stress on maize growth parameters (Krantev et al., 2008). Similarly, our results showed that the plant height, root length, and biomass of lettuce treated with 200 mg/L and 500 mg/L SA were significantly higher than those of Cd treatment alone (**Figure 1**). This indicated that exogenous SA could alleviate the growth inhibition of lettuce under Cd stress.

Cd stress can destroy the chloroplast ultrastructure of plant leaves and inhibit chlorophyll synthesis and leaf photosynthesis (Li et al., 2019b; Emanuil et al., 2020). The study showed that Cd stress significantly affected the photosynthetic pigment content, chlorophyll fluorescence (Fv/Fm), and photosynthetic parameters of the bean plant. When the bean was treated with 1.0 mmol/L SA, the Cd inhibition on the bean was significantly alleviated, and the photosynthetic parameters were significantly improved (Wael et al., 2015). Similarly, it was found that adding different concentrations of exogenous SA could effectively inhibit the reduction of photosynthetic pigment content (such as chlorophyll and carotenoid) caused by Cd stress and enhance the photosynthetic rate of leaves in *Iris hexagona* (Han et al., 2015) and *Lemna minor* (Lu et al., 2018). These findings are consistent with those reported in the present study. Under Cd stress, a certain concentration of SA increased the total chlorophyll and carotenoid contents of lettuce leaves, promoting photosynthesis (**Figure 2**, **Table 2**). The protective effect of SA on chlorophyll may be related to the alleviation of reduced chlorophyll enzyme activity caused by Cd stress and the enhancement of chlorophyll ester reductase activity related to chlorophyll synthesis (Kaur et al., 2017).

Many studies have shown that when plants are stressed by heavy metals, excessive production of related reactive oxygen species induces oxidative damage to plants (Su et al., 2020). An important mechanism by which SA alleviates Cd toxicity is strengthening the antioxidant defense system of the plant. This enables the plant to effectively prevent the excessive accumulation of ROS and slow down the oxidative damage caused by Cd stress (Li et al., 2019b; Liu et al., 2022). Furthermore, some studies showed that under Cd stress, SA pretreatment increased the content of antioxidant enzymes and non-enzyme antioxidants in maize, thereby reducing Cd-induced oxidative damage (Guo et al., 2009). Guo et al. reported that deleting SA in the SA-deficient mutant *sid2* of *Arabidopsis* aggravated the Cd-induced oxidative damage and growth inhibition (Guo et al., 2016). This study found that under Cd stress, a certain concentration of SA could reduce the relative conductivity, free proline content, and the POD and SOD activities of lettuce leaves (**Table 3**, **Figure 3**). Under Cd stress, SA treatment had varied effects on the activities of various antioxidant enzymes, possibly due to the different sensitivity levels of different plants or different tissues of the same plant to Cd and SA.

An important mechanism through which SA alleviates plant Cd toxicity is reducing plant uptake and transport of Cd (Singh et al., 2015; Gondor et al., 2016). Several studies showed that SA has a regulatory effect on the genes involved in Cd uptake and transport in plants, thus affecting the absorption of Cd ions (Jia et al., 2021). Singh et al. demonstrated that 100 μmol/L SA pretreatment could significantly increase the expression level of the natural resistance-associated macrophage protein 5 (*NRAMP5*) gene in rice seedling roots (Singh et al., 2015). At the same time, other studies have shown that ryegrass tolerance to Cd can be improved by increasing the expression of *Nramp2* in the stems and roots of ryegrasses (Li et al., 2019a). In addition to reducing the absorption and transport of Cd, other studies reported that SA could reduce Cd transportation from the aboveground parts to the grain, thus reducing Cd accumulation in the grain. Wang et al. reported that the Cd content in rice grains decreased from 0.29 mg · kg^-1^ to 0.12 mg · kg^-1^ after foliar application of 100 μmol/L SA (Wang et al., 2021d). The study also found that SA treatment could significantly reduce the Cd transfer from stem to leaf and from leaf to ear, possibly because Cd is mainly separated by the leaf cell vacuoles in leaves. This inhibits the expression of *OsLCT1* and *OsLCD* genes regulating Cd transport to rice grains, thus reducing Cd accumulation in grains (Wang et al., 2021d). The research results of Huang et al. showed that exogenous SA increased the expression of Cd stress tolerance genes (*OsHMA3* and *OsNRAMP5*) and Fe-transport-related genes (*OsIRT1*, *OsNRAMP1*, *OsNAS3*, and *OsYSL15*), thus enhancing the tolerance of rice to Cd and reducing the accumulation of Cd, Mn, and Fe (Huang et al., 2022a). Our research showed that under Cd stress, the total Cd content of lettuce increased initially and then decreased with the increase of exogenous SA concentration (**Figure 4**), similar to the research results of Wang et al. (2021c). In addition, our study also found that under Cd stress, a certain concentration of exogenous SA could up-regulate the relative expression of *HMA3*, *HMA4*, and *SAMT* (**Figure 5**). This was inconsistent with the expression trend of *HMA* reported by Chen et al. (2021) when exogenous gibberellin (GA) was used to alleviate Cd stress in lettuce, probably due to the different parts of lettuce used for foliar application or differences in action mechanisms of SA and GA. In general, being low-cost and eco-friendly, foliar SA application is one of the most economical and effective methods for reducing Cd accumulation by plants from Cd-contaminated soils.

## 5 Conclusion

The results showed that exogenous SA could alleviate the inhibitory effect of Cd stress on lettuce growth and promote the photosynthetic pigment content and parameters under Cd stress. SA could also effectively protect plant cells from oxidative damage by reducing the activities of osmoregulation substances and antioxidant enzymes induced by Cd stress. Additionally, a certain concentration of SA could downregulate the relative expression of *Nramp5*, *HMA4*, and *SAMT* genes, thereby reducing the Cd content in lettuce shoots. In summary, applying 100-500 mg/L SA could effectively reduce the Cd toxicity in lettuce and the Cd accumulation in the edible parts of lettuce.

## Data availability statement

The original contributions presented in the study are included in the article/**Supplementary Material**. Further inquiries can be directed to the corresponding author.

## Author contributions

WT, investigation and writing-original draft. LL, YX, XL, LJL, ZH, BS, GS, and LT, investigation. HL, conceptualization. YT, conceptualization, writing-review, and editing. All authors contributed to the article and approved the submitted version.
